# Transvaginal ultrasound cervical length for prediction of spontaneous labor at term: A protocol for systematic review and meta-analysis

**DOI:** 10.1097/MD.0000000000022237

**Published:** 2020-12-24

**Authors:** Lirong Wu, Gang Lei, Ming Tan

**Affiliations:** a Wuhan Hanyang Hospital; b Department of Obstetrics and Gynecology, Central Hospital of Wuhan, affiliated to Huazhong University of Science and Technology; c Hairdressing Department, Hubei Maternal and Child Health Hospital affiliated to Tongji Medical College of Huazhong University of Science and Technology, Wuhan City, Hubei Province, China.

**Keywords:** cervical length, labor, meta-analysis, transvaginal ultrasound

## Abstract

**Purpose:**

To evaluate the predictive accuracy of transvaginal ultrasound (TVU) cervical length (CL) for spontaneous onset of labor in singleton gestation enrolled at term by a meta-analysis.

**Materials and methods:**

This protocol established in this study has been reported following the Preferred Reporting Items for Systematic Review and Meta-Analysis Protocols. Web of Science, PubMed, EMBASE, and the Cochrane Library were searched for all clinical trials assessing the accuracy of TVU CL in prediction of spontaneous onset of labor in singleton gestations with vertex presentation who were enrolled at term until August 15, 2020. We will use a combination of Medical Subject Heading and free-text terms with various synonyms to search based on the eligibility criteria. Two investigators independently reviewed the included studies and extracted relevant data. The 95% confidence intervals (CIs) of was used as effect estimate. I-square (*I*^2^) test, substantial heterogeneity, sensitivity analysis, and publication bias assessment will be performed accordingly. Stata 15.0 and Review Manger 5.3 are used for meta-analysis and systematic review.

**Results:**

The results will be published in a peer-reviewed journal.

**Conclusion:**

The results of this review will be widely disseminated through peer-reviewed publications and conference presentations. This evidence may also assess the accuracy of TVU CL in prediction of spontaneous onset of labor in singleton gestations with vertex presentation.

**Registration number:**

INPLASY202080065

## 1 Introduction

The possibility to predict the delivery date is a question frequently raised by pregnant women. So far, data from the last menstrual period and the first accurate ultrasound examination are the 2 important methods for estimating gestational age and due date.^[1,2]^ Nonetheless, only 5% of women deliver on their due date.^[3]^ A clinician has currently little to predict when a woman at term, for example, 38 or 39 weeks, will deliver. Over the last few years, cervical assessment has moved from digital examination to ultrasound evaluation, and ultrasound of the cervix has been the focus of much research.^[4,5]^

Transvaginal ultrasound (TVU) cervical length (CL) has been assessed in several populations^[6,7]^ (e.g., asymptomatic women as well as women with symptoms of preterm labor) to evaluate the risk of preterm birth, and in women before induction of labor to predict induction outcome. Currently, many observational studies have evaluated the association between TVU CL at term and the interval to delivery.^[8]^ Although TVU CL is reproducible and easy to learn, studies demonstrate conflicting results regarding its predictive accuracy in this clinical scenario.^[9,10]^

Therefore, we plan do a systematic review and meta-analysis to evaluate the accuracy of TVU CL in the prediction of spontaneous onset of labor within 7 days in singleton gestations at term.

## 2 Study aim

The aim of our study is to evaluate the predictive accuracy of TVU CL for spontaneous onset of labor in singleton gestation enrolled at term.

## 3 Methods

The protocol of our meta-analysis followed the guideline of the Preferred Reporting Items for Systematic Review and Meta-Analysis Protocols (PRISMA-P) recommendations.^[11]^ It has been registered with International Prospective Register of Systematic Reviews (PROSPERO) as INPLASY database as INPLASY202080065 (https://inplasy.com/inplasy-2020-8-0065/).

### 3.1 Search strategy

A systematic search was performed in PubMed, Web of Science, Cochrane Library and Embase until August 15, 2020. The MeSH search and text word will be used with the terms related to “cervical length,” “delivery,” and “transvaginal ultrasound.” To perform a comprehensive and focused search, experienced systematic review researchers will be invited to develop a search strategy. An example of search strategy for PubMed database shown in Table 1 will be modified and used for the other databases. The reference lists of all relevant studies will be searched for additional relevant studies not retrieved from the electronic database search.

**Table 1:** Searching strategy in PubMed.

Serial number	Line
#1	“Cervical Length Measurement”[Mesh] OR “Cervical Length Measurements”[Title/Abstract] OR “Cervical Length”[Title/Abstract]
#2	“Delivery, Obstetric”[Mesh] OR “Deliveries, Obstetric”[Title/Abstract] OR “Obstetric Deliveries”[Title/Abstract] OR “Obstetric Delivery”[Title/Abstract]
#3	“Ultrasonography”[Mesh] OR “Diagnostic Ultrasound”[Title/Abstract] OR “Diagnostic Ultrasounds”[Title/Abstract] OR “Ultrasound, Diagnostic”[Title/Abstract] OR “Ultrasounds, Diagnostic”[Title/Abstract] OR “Ultrasound Imaging”[Title/Abstract] OR “Imaging, Ultrasound”[Title/Abstract] OR “Imagings, Ultrasound”[Title/Abstract] OR “Echotomography”[Title/Abstract] OR “Ultrasonic Imaging”[Title/Abstract] OR “Imaging, Ultrasonic”[Title/Abstract]
#4	#1 AND #2 AND #3

### 3.2 Eligibility criteria

Inclusion criteria: Studies were included if they reported data allowing construction of a 2 × 2 table. We included only studies assessing the accuracy of TVU CL in prediction of spontaneous onset of labor as defined by the authors, including spontaneous rupture of membranes, in singleton gestations with vertex presentation who were enrolled at term.

Exclusion criteria: Exclusion criteria included studies on women enrolled before 37 weeks or after 41 weeks, studies on women with premature rupture of membranes, studies on women with multiple gestations and case-report studies.

### 3.3 Study selection

**Figure 1:**
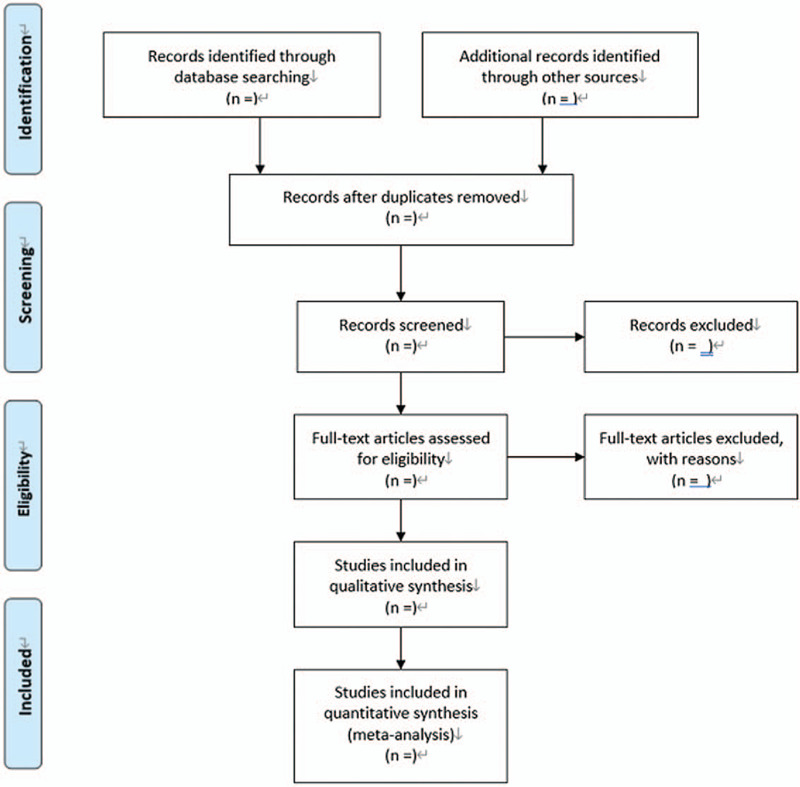
Flow diagram: selection process for the studies.

All initial records from 4 electronic databases will be imported into the web-based systematic review Rayyan software.^[12]^ First, the titles and abstracts of records will be reviewed independently by 2 reviewers to identify potential trials according to eligibility criteria. Then, full-text of all potentially relevant trials will be downloaded to make sure eligible trials. Any conflict will be resolved by discussion. A flow diagram (Fig. 1) will be used to describe the selection process of eligible papers.

### 3.4 Data extraction and management

The data will be extracted out by 2 independent reviewers in accordance with the Cochrane Handbook of Systematic Reviews of Interventions. Two investigators will independently screen all the included studies.

### 3.5 Risk of bias of individual study and quality assessment

Two reviewers will evaluate independently the risk of bias of included studies using a modified version of Cochrane tool^[13]^ in which we will to check for allocation concealment, blinding, incomplete outcome data, selective reporting, and other bias, each of which makes high risk, low-risk, and unclear grades. Any discrepancy was resolved by discussion or by a third reviewer.

### 3.6 Data analyses

For all the included studies we will construct a 2 × 2 table cross-classifying CL and the outcome of spontaneous onset of labor within 7 days using each CL measurement mentioned in the included studies. We will generate the Forest plot for the pooled sensitivity and specificity with 95% confidence interval (CI). A linear regression was performed to analyze the relation between CL (predictor variable; *X*) and the most important test characteristics (criterion variable; *Y*), that is, sensitivity, specificity, positive predictive value (PPV), and negative predictive value (NPV). Additionally, summary receiver-operating characteristics (sROC) curves will be plotted. The area under the curve (AUC) and the Q∗ index will be also computed to evaluate the overall performance of the diagnostic test accuracy. The AUC of an sROC curve is a measure of the overall performance of a diagnostic test in accurately differentiating those cases with and those without the condition of interest. The Q∗ index is defined by the point at which sensitivity and specificity are equal, which is closest to the ideal top-left corner of the sROC space. Both values range between 0 and 1, with higher values indicating better test performance. The following guidelines have been suggested for interpretation of AUC values: low (0.5 ≥ AUC < 0.7), moderate (0.7 ≥ AUC < 0.9), or high (0.9 ≥ AUC ≤ 1) accuracy.

### 3.7 Publication bias

If included studies were >10, funnel plot will be used to identify the possible publication bias. Additionally, Egg regression and Begg tests will be utilized to detect the funnel plot asymmetry.^[14]^

### 3.8 Subgroup analysis

If there is enough research, we will conduct a subgroup analysis to investigate differences in age, sex, and etc.

## 4 Discussion

Being able to predict the date of onset of spontaneous labor has several potential benefits.^[15]^ These data on TVU CL prediction of spontaneous labor may be helpful for women choosing between planned repeat caesarean delivery in the 39th week and awaiting spontaneous labor to attempt vaginal birth after a previous caesarean.^[16]^ It can enable better plans to be made regarding maternal transport; for example, a woman with a TVU CL of 10 mm at 37 weeks carrying a fetus with a congenital diaphragmatic hernia may want to move closer to the hospital if she is currently living far away.^[17]^ For pregnant women, this information may help them to arrange their social activities and deal with their anxiety. TVU CL as a screening test at term for prediction of spontaneous labor may be best considered in women who will benefit most from this test.^[18]^ Many previous studies^[8–10]^ have suggested that TVU CL might predict accurately for the spontaneous onset of labor in singleton gestation enrolled at term.

Thus, this systematic review and meta-analysis will evaluate the predictive accuracy of TVU CL for spontaneous onset of labor in singleton gestation enrolled at term. The results of this review will be widely disseminated through peer-reviewed publications and conference presentations. This evidence may also provide helpful evidence of whether TVU CL could accurately predict the spontaneous onset of labor in singleton gestations with vertex presentation.

## Author contributions

**Acquisition:** Lirong Wu, Gang Lei.

**Conceptualization:** Lirong Wu, Gang Lei, Ming Tan.

**Data curation:** Lirong Wu, Gang Lei, Ming Tan.

**Formal analysis:** Gang Lei, Ming Tan.

**Investigation:** Lirong Wu.

**Methodology:** Lirong Wu, Gang Lei, Ming Tan.

**Project administration:** Lirong Wu, Gang Lei, Ming Tan.

**Registration:** Ming Tan.

**Software:** Lirong Wu, Gang Lei.

**Supervision:** Lirong Wu, Gang Lei.

**Validation:** Lirong Wu, Gang Lei, Ming Tan.

**Visualization:** Ming Tan.

**Writing – original draft:** Lirong Wu, Gang Lei, Ming Tan.

**Writing – review & editing:** Ming Tan.
